# 1732. The Value of the Influenza Cell-Based Vaccine in the Pediatric Population. A Dynamic Transmission Modelling Approach in the U.S.

**DOI:** 10.1093/ofid/ofad500.1563

**Published:** 2023-11-27

**Authors:** Joaquin F Mould-Quevedo, Van Nguyen

**Affiliations:** CSL Seqirus Inc., Summit, New Jersey; VHN Consulting, Montreal, Quebec, Canada

## Abstract

**Background:**

Seasonal influenza poses a huge public health issue for children globally, resulting in significant disease and economic burdens. In addition, children are major influenza spreaders across the multiple age groups because they may pass on more of the virus for a longer period. This research aimed to evaluate the economic and health impacts of the cell-based quadrivalent influenza vaccine (QIVc) compared to the standard-dose egg-based quadrivalent influenza vaccine (QIVe) in U.S. children (6 months-17 years), from the payers and societal perspective.

**Methods:**

The public health and cost outcomes of QIVc and QIVe was estimated using a dynamic age-structured susceptible-exposed-infected-recovered transmission model. The model was calibrated to a low (2011-2012) and high incidence (2017-2018) influenza seasons, matching Centers for Disease Control and Prevention (CDC) reported data. The relative vaccine effectiveness of QIVc over QIVe in children was taken from a meta-analysis based on observational studies in the U.S. Within the dynamic model, adults (18-64yrs) received QIVc and older adults (65yrs+) the adjuvanted influenza vaccine; only vaccination type differences were accounted in the 6mons-17yrs age group. Probability of events, costs and utility values were obtained from U.S. literature. Coverage rates for all age-groups was taken from official CDC pre-pandemic reports. The robustness of the results was assessed in probabilistic sensitivity analyses.

**Results:**

Within a low incidence season (LIS), switching from QIVe to QIVc in 6 months- to 17-year-olds may prevent 2.9 million symptomatic cases, 1.1 million outpatient visits, 31,667 hospitalizations, and 4,163 deaths annually. As an opposite, within a high incidence season (HIS), switching from QIVe to QIVc may prevent 1.7 million symptomatic cases, 648,263 outpatient visits, 16,688 hospitalizations, and 2,126 deaths. In both scenarios, LIS and HIS, QIVc would be a cost-saving strategy with US$1.4B and US$468.7M savings from a societal perspective and US$989.8M and US$252.8M savings from a payer perspective, respectively.

**Conclusion:**

The analysis shows that QIVc in children is expected to prevent hospitalizations and deaths, and result in substantial savings in healthcare costs.

**Disclosures:**

**Joaquin F. Mould-Quevedo, PhD**, CSL Seqirus Inc.: Employee|CSL Seqirus Inc.: Employee|CSL Seqirus Inc.: Stocks/Bonds|CSL Seqirus Inc.: Stocks/Bonds **Van Nguyen, PhD**, CSL Seqirus Inc.: Advisor/Consultant|CSL Seqirus Inc.: Advisor/Consultant|CSL Seqirus Inc.: Honoraria

